# Public Views About Cosmetic Procedures in Riyadh, Saudi Arabia

**DOI:** 10.7759/cureus.50135

**Published:** 2023-12-07

**Authors:** Razan K Aldeham, Khalid Bin Abdulrahman, Sara K Habib, Lama M Alajlan, Malak K AlSugayer, Lana H Alabdulkarim

**Affiliations:** 1 College of Medicine, Imam Mohammad Ibn Saud Islamic University (IMSIU), Riyadh, SAU; 2 Department of Medical Education, Imam Mohammad Ibn Saud Islamic University (IMSIU), Riyadh, SAU

**Keywords:** demographic characteristics, saudi arabia, cosmetic surgeries in riyadh, public views, beauty standards, cosmetic procedures

## Abstract

Background

In the past decade, there has been a clear, massive increase in the number of patients undergoing cosmetic procedures in Riyadh, Saudi Arabia. This study aims to compare the demographic characteristics of patients in Riyadh undergoing cosmetic procedures.

Methodology

This is an analytical, observational, and cross-sectional study used to seek the public views about cosmetic procedures in Riyadh, Saudi Arabia using the snowball sampling technique. A self-administered questionnaire was distributed electronically among the target population via social media. The questionnaire was pre-tested in a pilot study of 10 individuals to ensure comprehension and ease of administration and to determine the time needed to complete it. The final adjustments were made after the pilot study. Statistical analysis was performed using SPSS Statistics version 25.0 (IBM Corp. Released 2017. IBM SPSS Statistics for Windows, Version 25.0. Armonk, NY: IBM Corp.).

Results

In the current study, we were able to collect the data from 600 participants, of whom females represented 527 (87.8%) of the sample. Satisfaction with their current physical appearance (370, 61.7%), financial factors (73, 12.2%), and fear of having the procedure (59, 9.8%) were the main reasons for refusing to undergo cosmetic procedures, while pleasing themselves (432, 72.1%), influence from others who have undergone cosmetic procedures (188, 31.4%), and personal dislike of one's appearance (184, 30.7%) were the main reasons for conducting cosmetic procedures. Firstly, gender showed a significant relationship with having undergone a cosmetic procedure (p-value = 0.018), with 9 (12.3%) of males and 131 (24.9%) of females reporting having undergone such a procedure. Age also played a role, with the 29-39 age group having the highest number of individuals (31.9%) who had undergone a cosmetic procedure.

Conclusion

This study provides valuable insights into the demographic characteristics, attitudes, and perceptions surrounding cosmetic procedures in Riyadh. The findings highlight the influence of cultural expectations, self-acceptance, social factors, and media on individuals' motivations and decision-making processes. The increasing acceptability of cosmetic interventions and the rising demand for aesthetic modifications in the locality suggest a shifting societal landscape.

## Introduction

Recently, there has been increasing demand in the discipline of cosmetic surgery [1]. Plastic surgery is a surgical specialty involving the restoration, reconstruction, or alteration of the human body. It can be divided into two main categories: reconstructive surgery and cosmetic surgery [2,3]. Reconstructive surgery includes craniofacial surgery, hand surgery, microsurgery, and the treatment of burns [4]. Throughout history, beauty standards have evolved from era to era to the extent that what is considered “beautiful" today was once disapproved by society [5,6]. Without a doubt, individuals strive to meet beauty standards set by society regardless of whether they personally believe in them. With advancements in medicine and technology, cosmetic procedures have become a tool for individuals to “fit in” and meet the beauty criteria set by society [7]. Keeping this in mind, it is increasingly critical to understand the public’s view towards such procedures. In consonance with a study performed by the International Society of Aesthetic Plastic Surgery, Saudi Arabia holds the 22nd position among the top 25 countries by total number of procedures [1]. In addition, according to the American Society of Plastic Surgeons' annual plastic surgery statistics report, there were more than 17.7 million surgical and minimally invasive cosmetic procedures conducted in the United States in 2018, a number that has risen steadily over the past five years [2]. A cross-sectional study conducted by researchers in Riyadh, Saudi Arabia, using convenient sampling on 500 females at different venues (participants were given a questionnaire) found that 277 (55.4%) of them had done a cosmetic procedure. Among the participants, 273 (54.7%) had no cosmetic surgery because they did not need it, while 85 (17%) of them said no because of financial reasons and 47 (9.4%) because of social causes. Among those who underwent cosmetic surgery, 160 (58.1%) responded that their purpose was to change and have a more beautiful look. However, 86 (31%) of them performed cosmetic surgeries to reverse changes that occurred to their faces [1]. In a study by Furnham and Levitas, a sample of 204 British participants were asked to fill in a questionnaire that evaluated their attitude towards cosmetic procedures and measured their self-esteem, religiosity, and satisfaction with their current life standards [3]. In addition, the extent the participant watched the media was also considered. Females with low self-esteem, poor life satisfaction, low self-rated attractiveness, and limited religious beliefs who were heavy media watchers reported a greater likelihood of undergoing cosmetic surgery as compared to others [3]. This study highlights many of the important causative factors patients consider before thinking about cosmetic procedures.

## Materials and methods

Study design

This study employed an analytical, observational, and cross-sectional design to investigate the public views about cosmetic procedures in Riyadh, Saudi Arabia. A self-administered questionnaire was distributed electronically among the target population via social media. The questionnaire was pre-tested in a pilot study of 10 individuals to ensure comprehension and ease of administration and to determine the time needed to complete it. The final adjustments were made after the pilot study. Statistical analysis was performed using SPSS Statistics version 25.0 (IBM Corp. Released 2017. IBM SPSS Statistics for Windows, Version 25.0. Armonk, NY: IBM Corp.). The study adhered to ethical guidelines and obtained informed consent from participants.

Sampling technique

Snowball sampling was utilized to recruit participants for this study. The initial participants were selected purposefully based on their availability and willingness to participate. They were then asked to refer other potential participants from their social networks who met the inclusion criteria. This chain referral method allowed for a wider reach and a greater diversity of participants.

Data collection

An online questionnaire was developed and distributed using social media platforms to reach the target population in Riyadh. The questionnaire consisted of items related to public views on cosmetic procedures, including their perceptions, knowledge, attitudes, and experiences. Participants were able to complete the questionnaire at their convenience, ensuring flexibility and wider participation.

Inclusion criteria

The study included individuals from the general population of Riyadh city, both Saudi citizens and other Riyadh residents, aged between 18 and 65 years. This age range was selected to capture a broad representation of adults who may have varying perspectives on cosmetic procedures.

Exclusion criteria

There were no specific exclusion criteria for participation in this study. Once individuals met the inclusion criteria and voluntarily participated by completing the questionnaire, they were included in the analysis.

Data analysis

Upon completion of data collection, the collected data were organized and entered into the SPSS software for analysis. Descriptive statistics were used to summarize the data, including frequencies and percentages. Categorical variables were presented as frequencies and percentages to provide a clear understanding of participants' views on cosmetic procedures. The results were visually presented using bar charts and tables to facilitate data interpretation.

Ethical considerations

This study adhered to ethical guidelines to protect the rights and confidentiality of participants. Informed consent was obtained from all participants before they participated in the study. The questionnaire ensured anonymity and confidentiality by not collecting any personally identifiable information. The study also complied with relevant data protection regulations and guidelines. The study was approved by the Institutional Research Board at Imam Mohammed Ibn Saud Islamic University (IMSIU) (HAPO-01-R-0011). All study participants were informed of the study’s objectives and were assured that their responses would be kept confidential (IRB approval number: 550/2023).

## Results

Table 1 presents the demographic characteristics of the participants (N=600). The majority of participants were female, making up 527 participants (87.8%), while males accounted for 73 participants (12.2%) of the sample. In terms of age, the largest proportion of participants, 333 (55.5%), fell within the 18-28 age group, followed by 122 (20.3%) in the 40-50 age group. Saudi participants constituted the majority, 588 (98.0%), with a small percentage of non-Saudi participants, 12 (2.0%). Regarding previous cosmetic procedures, 460 (76.7%) participants reported never having undergone any cosmetic procedures.

**Table 1:** Demographic factors of the participants

Demographic characteristics		Number of participants	(%)
Gender	Male	73	12.2%
	Female	527	87.8%
Age	18-28	333	55.5%
	29-39	47	7.8%
	40-50	122	20.3%
	51-65	98	16.3%
Nationality	Saudi	588	98.0%
	Not-Saudi	12	2.0%
Have you ever had a cosmetic procedure?	No	460	76.7%
	Yes	140	23.3%

**Figure 1:**
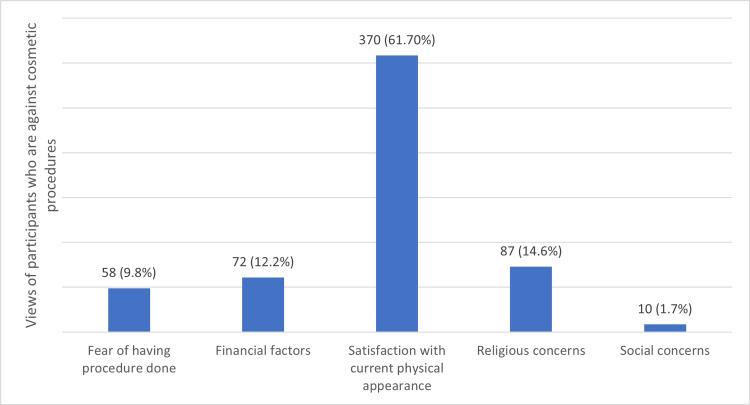
Why you haven't had or considered having cosmetic surgery?

Figure 1 illustrates the reasons why participants reported not having or considering cosmetic surgery. The most common reason was satisfaction with their current physical appearance, with 370 (61.7%) of participants selecting this option. Financial factors were cited by 72 (12.2%) of participants, while fear of having the procedure done was chosen by 58 (9.8%) of participants. Religious concerns were reported by 87 (14.6%) of participants, and social concerns were mentioned by a smaller number of 10 participants (1.7%).

**Figure 2:**
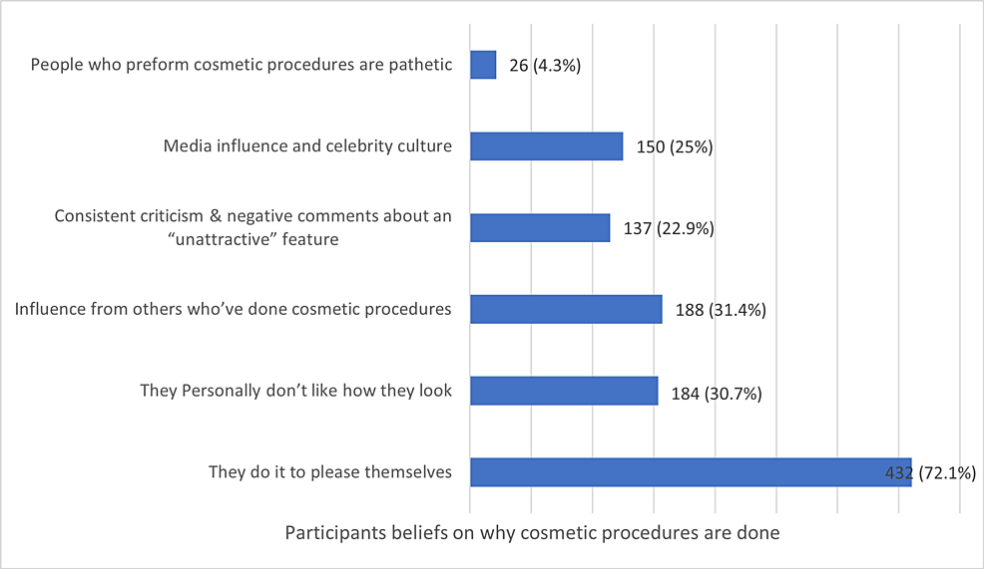
Why do you think people have cosmetic procedures done?

Figure 2 displays the reasons participants believed people had cosmetic procedures done. The most prominent reason was to please themselves, selected by 432 (72.1%) of participants. The influence of others who have undergone cosmetic procedures was cited by 188 (31.4%) of participants, while personal dislike of one's appearance was chosen by 184 (30.7%) of participants. Consistent criticism and negative comments about an "unattractive" feature were mentioned by 137 (22.9%) of participants, and media influence and celebrity culture were reported by 150 (25%) of participants. A small number of participants, 26 (4.3%), believed that people who perform cosmetic procedures are pathetic.

Table 2 provides insights into the attitudes of the participants towards cosmetic procedures. The majority of participants, 385 (64.2%), reported that they would tell others if they had undergone a cosmetic procedure, while 215 (35.8%) stated they would keep it a secret. Regarding the acceptability of cosmetic procedures based on age, a significant proportion, 408 (68.0%), believed that age does not matter, indicating that cosmetic procedures are acceptable across different age groups. In terms of gender, nearly half of the participants, 296 (49.4%), believed that gender does not matter when it comes to the acceptability of cosmetic procedures. When asked if they would encourage a friend or relative with an unattractive feature to undergo a cosmetic procedure, 212 (35.3%) responded affirmatively. Additionally, a substantial portion of participants, 259 (43.2%), believed that individuals would be happier and more confident if they underwent a cosmetic procedure to address an unattractive feature. Concerning the perception of cosmetic procedure rates in Riyadh, the majority of participants, 505 (84.2%), believed that rates have been increasing. Furthermore, a significant proportion of participants, 366 (61.0%), perceived that cosmetic procedures are more acceptable nowadays in Riyadh. In terms of societal attitudes towards natural appearance, a larger percentage, 268 (44.7%), felt that people have become more critical in the past five years. Regarding awareness of complications, the majority of participants, 398 (66.3%), reported being aware of the potential complications associated with different cosmetic procedures, while a smaller proportion of participants, 54 (9.0%), believed that cosmetic procedures were completely safe.

**Table 2:** The attitude of participants towards cosmetic procedures

Questions asked to reflect participants' attitudes towards cosmetic procedures		Number of participants	(%)
After performing a cosmetic procedure, would you tell others or keep it a secret?	Tell others	385	64.2%
	Keep it a secret	215	35.8%
Do you think cosmetic procedures are acceptable only for a certain age group?	Age does not matter	408	68.0%
	Young people only	27	4.5%
	Middle-aged and elderly only	165	27.5%
Do you think cosmetic procedures are acceptable only for a certain gender?	Gender does not matter	296	49.4%
	For both	154	25.7%
	Females only	149	24.9%
If a friend or relative had a feature that was considered unattractive in society, would you encourage them to perform a cosmetic procedure?	No	388	64.7%
	Yes	212	35.3%
If a friend or relative had a feature that was considered unattractive in society, do you think he/ she would be happier and more confident if they performed a cosmetic procedure?	No	36	6.0%
	Yes	259	43.2%
	I do not think there is any relationship between happiness/ confidence and cosmetic procedures	305	50.8%
Do you think cosmetic procedure rates are increasing in Riyadh?	No	4	0.7%
	Yes	505	84.2%
	I do not know	91	15.2%
Do you think cosmetic procedures are more acceptable nowadays in Riyadh?	No	0	0.0%
	Yes	366	61.0%
	I do not know	84	14.0%
	Maybe	150	25.0%
Do you think people have been more or less critical about the natural appearance of others in the past 5 years?	No change	202	33.7%
	More critical	268	44.7%
	Less critical	129	21.5%
Are you aware of any of the complications of different cosmetic procedures?	No	54	9.0%
	Yes	398	66.3%
	Cosmetic procedures are completely safe	9	1.5%
	I do not know	139	23.2%

Table 3 provides insights into the relationship between demographic factors and the experience of having a cosmetic procedure. The results revealed significant associations in several areas. Firstly, gender showed a significant relationship with having undergone a cosmetic procedure (p-value = 0.018), with nine (12.3%) males and 131 (24.9%) females reporting having undergone such a procedure. Age also played a role, with the 29-39 age group having the highest number, 15 (31.9%) of individuals who had undergone a cosmetic procedure. Nationality did not show a significant association with undergoing cosmetic procedures, as both Saudi and non-Saudi participants had similar proportions. Regarding perceptions of criticality towards natural appearance, no significant relationship was found. However, awareness of complications associated with cosmetic procedures showed a significant association (p-value = 0.041*), with 98 (24.6%) who were aware of complications having a higher likelihood of having undergone a cosmetic procedure.

**Table 3:** The relationship between demographic factors and having a cosmetic procedure

Demographic features		Have you ever had a cosmetic procedure?				
		No		Yes		p-value
		Number of participants	(%)	Number of participants	(%)	
Gender	Male	64	87.7%	9	12.3%	0.018*
	Female	396	75.1%	131	24.9%	
Age	18-28	267	80.2%	66	19.8%	0.046*
	29-39	32	68.1%	15	31.9%	
	40-50	94	77.0%	28	23.0%	
	51-65	67	68.4%	31	31.6%	
Nationality	Saudi	451	76.7%	137	23.3%	0.890
	Not-Saudi	9	75.0%	3	25.0%	
Do you think people have been more or less critical about the natural appearance of others in the past 5 years?	No change	159	78.7%	43	21.3%	0.113
	More critical	195	72.8%	73	27.2%	
	Less critical	105	81.4%	24	18.6%	
Are you aware of any of the complications of different cosmetic procedures?	No	46	85.2%	8	14.8%	0.041*
	Yes	300	75.4%	98	24.6%	
	Cosmetic procedures are completely safe	4	44.4%	5	55.6%	
	I do not know	110	79.1%	29	20.9%	

## Discussion

The primary objective of the current study was to examine the demographic characteristics, attitudes, and views pertaining to cosmetic operations among the participants. The results of this study offer significant contributions to our understanding of the determinants that shape individuals' decision-making processes and contribute to our knowledge of social views regarding cosmetic operations in Riyadh.

The participants' demographic data indicated a primarily female sample where the sample consisted of a majority of females, making up 527 (87.8%) of the total, which aligns with the greater occurrence of cosmetic procedures among women [8]. The aforementioned discovery aligns with the cultural milieu of Saudi Arabia, wherein women may encounter heightened societal expectations to adhere to specific ideals of physical attractiveness [9]. The greater proportion of women seeking cosmetic operations can be ascribed to cultural pressures that prioritize appearance and the enhancement of beauty for women [7,10].

In relation to the distribution of age, the observation that the age cohort ranging from 29 to 39 years exhibited the greatest proportion of persons who opted for cosmetic interventions is consistent with other scholarly investigations that have suggested a higher propensity for individuals in their late twenties to early forties to contemplate aesthetic modifications [3,5].

In addition, the current study illustrates that a significant proportion of participants expressed contentment with their present physical appearance, which emerged as the primary rationale behind their decision to abstain from or not contemplate undergoing cosmetic surgery. The aforementioned discovery aligns with other research that suggests a notable fraction of persons who choose not to have cosmetic operations are satisfied with their inherent physical appearance [11-13]. This implies that the concepts of self-acceptance and body positivity have an influence on individuals' choices pertaining to cosmetic modifications.

A minority of individuals cited financial considerations as a deterrent to pursuing cosmetic surgery. The aforementioned discovery aligns with other scholarly investigations that have recognized cost as a significant obstacle in the realm of accessing cosmetic operations [14]. The financial implications associated with cosmetic operations can provide a substantial barrier for certain individuals, particularly when considering the possible costs associated with several sessions or more invasive interventions [15].

Moreover, the study offers valuable insights into the rationales for participants' perceptions of the motivations for individuals seeking cosmetic procedures. The primary factor, selected by a majority of respondents, was personal gratification. The aforementioned study conducted by Al Ghadeer et al. indicated that individuals' pursuit of cosmetic operations is driven by the need for self-enhancement and personal happiness [16]. This perspective is in accordance with the notion of self-enhancement and the aspiration to conform to societal standards of beauty as subjective decisions and manifestations of personal agency.

A considerable proportion of participants acknowledged the impact of individuals who have previously undergone cosmetic operations. This discovery provides more support for other studies that suggest the importance of social factors and endorsements from acquaintances, relatives, or public figures in influencing individuals' choices to pursue cosmetic modifications [7,17-19]. The proposition posits that social elements, such as interpersonal connections and social comparisons, have the potential to impact individuals' perceptions and motivations in regard to cosmetic operations [20]. Motivations for undergoing cosmetic operations were found to include personal dissatisfaction with one's physical appearance and the presence of persistent criticism or harsh remarks over a perceived "unattractive" attribute. The results of this study align with the psychological aspects linked to body dissatisfaction and the aspiration for cosmetic modifications [6,21].

A significant proportion of individuals indicated the impact of media and celebrity culture. The aforementioned discovery aligns with previous research that highlights the influence of media and cultural ideals of beauty on individuals' perceptions of their own bodies and aspirations for cosmetic procedures [7,22]. The proposition posits that media depictions and the sway of celebrities have the capacity to mold individuals' conceptions of attractiveness and play a role in their impetus to pursue cosmetic interventions [3,7].

The individuals' attitudes towards aesthetic operations yielded intriguing information. The findings indicate that a significant proportion of participants expressed their willingness to divulge information about having undergone a cosmetic operation to individuals in their social circles. The aforementioned discovery aligns with other scholarly investigations that have demonstrated a pattern of increased acceptance and diminished social disapproval surrounding cosmetic interventions [23,24]. This observation indicates an increasing level of societal acceptability and normality of cosmetic modifications, which has created an environment where individuals feel comfortable openly discussing their own experiences with such procedures.

In relation to the acceptability of cosmetic procedures in relation to age and gender, a substantial number of respondents expressed the viewpoint that age and gender should not serve as restrictive criteria. The aforementioned discovery suggests a transition towards a viewpoint that is more encompassing and equitable towards cosmetic operations, which is in line with current societal principles of self-expression and independence [25,26]. Nevertheless, it is crucial to take into account that public attitudes could fluctuate across diverse cultural contexts, and additional investigation is required to delve deeper into these processes.

A prevailing consensus among participants was that there has been a notable rise in rates of cosmetic procedures, indicative of an escalating desire for aesthetic modifications within the locality. This discovery is consistent with global patterns that demonstrate an increase in the prevalence of cosmetic treatments [27,28]. Furthermore, a significant number of respondents held the perception that cosmetic operations are experiencing a rise in acceptance within the city of Riyadh.

The results of this study are consistent with prior studies about the demographic attributes, attitudes, and perceptions associated with cosmetic procedures. The frequency of female participants and the greater occurrence of cosmetic procedures among women align with other research undertaken in diverse cultural settings [27,29-31]. The findings of this study align with previous scholarly works that suggest a higher propensity among younger individuals to pursue cosmetic interventions, potentially attributable to societal expectations and the impact of social media [32].

This study had some limitations, as it is crucial to acknowledge that the conclusions of the research are limited to the particular setting of Riyadh and may not be applicable to other areas in Saudi Arabia or diverse cultural environments. Cultural variables, cultural standards, and aesthetic ideals exhibit variability across different areas and countries, necessitating further investigation to delve into these dynamics more comprehensively.

## Conclusions

This study makes a valuable contribution to the current body of literature by offering new insights into the demographic attributes, attitudes, and perceptions associated with cosmetic treatments in the city of Riyadh. The results underscore the necessity of acquiring a thorough comprehension of the various aspects that impact individuals' decision-making processes and society's attitudes regarding cosmetic modifications. Subsequent investigations may expand upon these findings in order to delve deeper into cultural disparities, psychological incentives, and enduring consequences linked to aesthetic interventions.
